# A TP53-Associated Immune Prognostic Signature for the Prediction of Overall Survival and Therapeutic Responses in Muscle-Invasive Bladder Cancer

**DOI:** 10.3389/fimmu.2020.590618

**Published:** 2020-12-17

**Authors:** Xiangkun Wu, Daojun Lv, Chao Cai, Zhijian Zhao, Ming Wang, Wenzhe Chen, Yongda Liu

**Affiliations:** ^1^ Department of Urology, Minimally Invasive Surgery Center, The First Affiliated Hospital of Guangzhou Medical University, Guangzhou, China; ^2^ Guangdong Key Laboratory of Urology, Guangzhou Institute of Urology, Guangzhou, China

**Keywords:** muscle-invasive bladder cancer, TP53 mutation, immune prognostic signature, nomogram, the cancer genome atlas (TCGA) and gene expression omnibus (GEO) database

## Abstract

**Background:**

TP53 gene mutation is one of the most common mutations in human bladder cancer (BC) and has been implicated in the progression and prognosis of BC.

**Methods:**

RNA sequencing data and TP53 mutation data in different populations and platforms were downloaded from The Cancer Genome Atlas (TCGA) and Gene Expression Omnibus (GEO) database to determine and validate a TP53-associated immune prognostic signature (TIPS) based on differentially expressed immune-related genes (DEIGs) between muscle-invasive bladder cancer (MIBC) patients with and without TP53 mutations.

**Results:**

A total of 99 DEIGs were identified based on TP53 mutation status. TIPS including ORM1, PTHLH, and CTSE were developed and validated to identify high-risk prognostic group who had a poorer prognosis than low-risk prognostic group in TCGA and GEO database. The high-risk prognostic group were characterized by a higher abundance of regulatory T cells, myeloid-derived suppressor cells, and tumor-associated macrophages than the low-risk prognostic group. Moreover, they exhibited a lower abundance of CD56bright NK cells, higher expression of CTLA4, LAG3, PDCD1, TIGIT, and HAVCR2, as well as being more likely to respond to anti–PD-1, and neoadjuvant chemotherapy than the low-risk prognostic group. Based on TIPS and other clinical characteristics, a nomogram was constructed for clinical use.

**Conclusion:**

TIPS derived from TP53 mutation status is a potential prognostic signature or therapeutic target but additional prospective studies are necessary to confirm this potential.

## Introduction

Bladder cancer (BC) is one of the most prevalent urothelial tumors and the leading cause of morbidity and mortality globally (1). Out of all the BC patients, about 25% are diagnosed with muscle-invasive bladder cancer (MIBC), which is characterized by rapid progression, metastasis, and poor prognosis (2). There are two different subtypes of MIBC, i.e., intrinsic basal and luminal subtypes (3). Reports have shown that MIBC is the more aggressive type, matched with lymph-node metastases and associated with a poorer survival rate (4). Increasing lines of evidence support the idea that the malignant phenotype of tumors is related to the tumor microenvironment (TME) (5, 6). Notably, MIBC is an immune-sensitive malignancy with multiple tumor-infiltrating lymphocytes (TILs), including, regulatory T cells (Tregs), myeloid-derived suppressor cells (MDSCs) and tumor-associated macrophages (TAMs) (7, 8). Therefore, advancements in detection, therapy, and prognosis of BC necessitate the integration of various new methods, including, genomic profiling, development of biomarkers, and immunotherapy (4). However, only a few studies have systematically investigated the association between the immunophenotype of TME and the prognosis of MIBC.

As the most studied tumor suppressor gene, TP53 (P53) is one of the most common mutated genes in human cancer (9). TP53 gene functions by binding directly to chromatin, it can sense cellular stress or damage, which in turn triggers cell apoptosis after DNA damage or causes cell cycle arrest (10, 11). Nonetheless, when the TP53 gene is mutated (mostly missense), it loses its function as a tumor suppressor gene and simultaneously promotes tumorigenesis (12). Subsequently, cells escape from DNA damage resulting in the unlimited proliferation of tumor cells and ultimately causing cancer (12). Germline mutations in TP53 induce a rare high penetrance cancer syndrome (13). A pan-cancer analysis of the frequency of DNA alterations across cell cycle activity levels revealed that TP53 mutations were prevalent in all cell cycle scores (14). Also, it has been argued that the high mutation rate of the TP53 gene is a potential target for gene therapy (15). Currently, molecular strategies of drugs targeting TP53 mutations are undergoing clinical trials (16). It was reported that TP53 genes are the most common drivers of mutations in MIBC (17, 18), also, BC patients with TP53 mutation displayed a shorter overall survival (OS) (18, 19, 20). As a result, it is fundamental to precisely explore the role of the TP53 gene in the pathogenesis of MIBC.

Interestingly, several recent studies reported that TP53 gene mutation property is closely linked to TME as well as different immune responses in multiple tumors (21–23). Therefore, we conjectured that the poor prognosis of MIBC patients with TP53 mutations can potentially be associated with TME. In this study, a comprehensive analysis of TP53 gene mutation and expression was performed to explore the association between TP53 mutations and immune phenotype in MIBC. Differentially expressed immune-related genes (DEIGs) were explored in patients with TP53 mutations (TP53^mut^) and without TP53 mutations (TP53^wt^). Importantly, we developed a TP53-associated immune prognostic signature (TIPS) with immune-related genes whose expression was affected by TP53 mutation, which has been confirmed to be a reliable biomarker and predictor of prognosis.

## Materials and Methods

### RNA Sequencing Data

The RNA sequencing data (HTSeq-Counts), MuTect2-based somatic mutation data, and the corresponding clinical data of 412 urinary bladder cancer patients were downloaded from The Cancer Genome Atlas (TCGA) website (https://portal.gdc.cancer.gov/). The selection criteria of these patients included; (1) MIBC tissue samples; (2) having TP53 mutation data; (3) having gene expression data; (4) having follow-up data and survival status. A total of 402 MIBC patients were enrolled in the TCGA cohort. Then, HTSeq-Counts data were converted to transcripts per kilobase million (TPM) data and were log2-transformed (log2TPM) for subsequent analysis, which showed more comparability between samples (24). This study used the average expression value of genes in instances where gene duplication was detected.

### Microarray Data

The matrix files of the gene expression profile and clinical information of GSE32894, GSE48075, and GSE52219 data sets were extracted from the Gene Expression Omnibus (GEO) database (https://www.ncbi.nlm.nih.gov/geo/). Gene expression data of GSE48075 was log2-transformed. Notably, the average gene expression was used if multiple probes matched a single gene. Batch effects were removed through the “sva” package (version: 3.36.0; http://bioconductor.org/packages/release/bioc/html/sva.html). Then, GSE32894 (MIBC patients: 51) and GSE48075 (MIBC patients: 73) based on the same platform GPL6947 with follow-up data and survival status were integrated into the GEO cohort (n = 124). Twenty-three MIBC patients with information regarding their response to neoadjuvant chemotherapy (NAC) (NAC type: MVAC=methotrexate, vinblastine, adriamycin, and cisplatin) in the GSE52219 data set were included into this study. Data from the present study are publicly available from The Cancer Genome Atlas (TCGA) and GEO databases.

### Gene Set Enrichment Analysis of TP53 Mutation

To identify potential differences in immunological pathways between TP53^mut^ (n=196) and TP53^wt^ (n=212) MIBC patients, GSEA (Version: 4.0; http://software.broadinstitute.org/gsea/index.jsp) was performed in TCGA cohort based on the reference gene set “c5.bp.v7.1.symbols.gmt.” The threshold was set at |Normalized Enrichment Score (NES)| > 1 and *P* < 0.01.

### Association Between the TP53 Mutation and Tumor Mutation Burden

TMB is defined as the total number of deletions, insertions, base substitutions, or somatic gene coding errors detected per million bases. To explore the association between TP53 mutation and TMB, Perl scripts (version 5.30.2; http://strawberryperl.com/) were used to calculate the mutation rate with number of variants/the length of exons (38 million) for each sample of the TCGA cohort based on MuTect2-based somatic mutation data.

### Differentially Expressed Immune-Related Genes Analysis

To identify DEIGs between TP53^mut^ (n=196) and TP53^wt^ (n=212) MIBC patients in the TCGA cohort, the “edgeR” package (version 3.30.0; http://www.bioconductor.org/packages/release/bioc/html/edgeR.html) was used with thresholds being false discovery rate (FDR) < 0.05 and |log2-fold change (FC)| > 1.0. The list of immune-related genes was downloaded from the ImmPort database (http://www.immport.org), which contains 17 immune categories based on different molecular function, such as TNF family receptors, B cell receptor signaling pathway, T cell receptor signaling pathway, and cytokines (25).

### Function Enrichment Analysis of Differentially Expressed Immune-Related Genes

Gene Ontology (GO) enrichment analysis, including biological processes (BP), cellular components (CC), and molecular functions (MF), and Kyoto Encyclopedia of Genes and Genomes (KEGG) pathway analyses were conducted using “clusterProfiler” package (version 3.16.1; http://www.bioconductor.org/packages/release/bioc/html/clusterProfiler.html) (26).

### Development and Validation of TP53-Associated Immune Prognostic Signature for Muscle-Invasive Bladder Cancer

Univariate Cox regression analysis was performed using “survival” package (version 3.1-12; https://cran.r-project.org/web/packages/survival/index.html) to assess the relationship between DEIGs and OS of MIBC patients in the TCGA cohort. DEIGs with *P* < 0.05 were considered prognostic immune-related genes. Further, the least absolute shrinkage and selection operator (LASSO) analysis was performed using the “glmnet” package (Version: 4.0; https://cran.r-project.org/web/packages/glmnet/index.html) to further screen prognostic DEIGs, which can resolve the collinearity problem and overfitting problem. Finally, TIPS was constructed by a multivariable Cox regression model based on the result from LASSO analysis, and the risk score of each patient was calculated by weighted estimators corresponding to the expression level of each gene. The patients in the TCGA cohort were divided into low- and high-risk prognostic groups as per the best cut off value determined by X-tile 3.6.1 software. To obtain the same formula and the uniform cutoff value to divide MIBC patients into low- and high-risk prognostic groups, a normalization for expression values of TIPS genes was conducted in the TCGA cohort and the GEO cohort with standard deviation (SD) = 1 and mean value = 0. To validate the TIPS, the risk score for each patient of the GEO cohort was calculated using the same formula and the patients were stratified into low- and high-risk prognostic groups following the same cutoff value obtained from the TCGA cohort. Of note, Kaplan-Meier survival analysis with the log-rank test was used to evaluate the survival difference between the low- and high-risk prognostic groups. Area under the curve (AUC) of time-dependent receiver operating characteristic (tROC) curve and the discrimination was used to evaluate prognostic performance of TIPS. The discrimination of TIPS was determined by a concordance index (C-index) using 1000 bootstrap resamples.

### Gene Set Enrichment Analysis of the Tp53-Associated Immune Prognostic Signature Genes

GSEA was performed to explore the enriched KEGG pathways of the TIPS genes. According to the median expression of each gene, 408 MIBC samples were divided into low- and high-expression groups. The gene set “c2.cp.kegg.v7.1.symbols.gmt” was chosen as the reference set, which was obtained from the Molecular Signatures Database V7.1 (MSigDB). |NES| > 1 and *P* < 0.01 were considered as statistically significant.

### Estimation of Tumor-Infiltrating Lymphocytes Abundance

To investigate the association between TIPS and TILs, single sample gene set enrichment analysis (ssGSEA) was performed to determine the relative abundance of 28 subpopulations of TILs in the MIBC immune infiltrates, including Tregs, MDSC, TAMs, CD56^bright^ NK cells (NK) cells, etc (27, 28).

### Correlation Between Tp53-Associated Immune Prognostic Signature and Biomarkers for Immunotherapy

Notably, the immune checkpoints are the biomarkers for selecting patients with MIBC for immunotherapy. Therefore, this study analyzed the correlation between TIPS and critical immune checkpoints (PD1, CTLA4, LAG3, HAVCR2, and TIGIT).

### Prediction of Chemotherapeutic and Immunotherapeutic Response

Chemotherapy is effective for treating MIBC. Based on the Genomics of Drug Sensitivity in Cancer (GDSC) website, the clinical response to chemotherapy of each MIBC patient was estimated to explore whether there were differences in the response of low- and high-risk prognostic groups to chemotherapy and immunotherapy. Two commonly used chemotherapy drugs, gemcitabine and cisplatin, were selected for the chemotherapeutic response prediction, which involved the ridge regression based on the “pRRophetic” R package (https://github.com/paulgeeleher/pRRophetic20) to predict the half-maximal inhibitory concentration (IC50) of each TCGA-MIBC patient and the 10-fold cross-validation based on the GDSC training set was used to assess the prediction accuracy (29). To validate the feasibility of the TIPS to predict response to NAC, 23 MIBC patients with NAC in GSE52219 data set were included into this study. A normalization for the expression of TIPS genes was conducted in GSE52219 data set with a standard deviation (SD) = 1 and mean value = 0. The risk score for each patient was calculated using the same formula.

The immune checkpoint inhibitor has evolved to be the most potent tool for cancer therapy, such as anti-PD1 and anti-CTLA4, which activate the immune system to play an anti-tumor role (30). Here, subclass mapping on GenePattern website (https://cloud.genepattern.org/gp) was used to predict the anti-PD1 and anti-CTLA4 response of each MIBC patient as described previously (31, 32).

### Independence of Tp53-Associated Immune Prognostic Signature From Clinical Characteristics

Out of the 402 MIBC patients in the TCGA cohort with follow-up and survival status data, 342 MIBC patients with complete clinical data, including age, gender, T stage, N status and TMB, were selected for subsequent analyses. Univariate and multivariate Cox regression analysis was performed to identify whether the prediction of TIPS was independent from other clinical characteristics (age, gender, T stage, N status and TMB). To further identify whether the TIPS was a statistically significant prognostic factor regardless of clinical data, the patients were stratified based on age, T stage and TMB, and Kaplan-Meier survival analysis was used to assess the difference in the OS between low- and high-risk prognostic groups of different subgroups.

### Development and Evaluation of the Nomogram Based on the Tp53-Associated Immune Prognostic Signature

To facilitate the prediction of 1-, 3-, and 5-year OS probability in MIBC patients, a nomogram was developed based on the results from multivariate Cox regression analyses using the “rms” R package (version 6.0-0; https://cran.r-project.org/web/packages/rms/index.html). The commonly used methods i.e., AUC of tROC, C-index using 1000 bootstrap resamples, and calibration plot were used to validate the performance of the nomogram. Calibration plots were used to assess the consistency between nomogram-predicted probabilities and observed probabilities using 500 bootstrap resamples, with the 45degree line representing the ideal predicted values. Moreover, decision curve analysis (DCA) was used to determine the net benefits derived from the use of the nomogram, TIPS, age, T stage, N status and TMB (33).

### Statistical Analysis

The 95% confidence interval (CI) and HR (Hazard Ratio) were generated by Cox regression analysis and Kaplan-Meier survival analysis. Cox regression analysis assume that the HR is constant over time, therefore, is significant to assess the validity of the proportional hazards assumption (PH assumption). The scale Schoenfelder residual test based on the “survival” R package (Version 3.2-7; https://cran.r-project.org/web/packages/survival/index.html) was performed to evaluate whether clinical variables violated the PH assumption (34). The FDR method was used to control for multiple testing, which is the percentage of important tests that can lead to a false positive (35). Spearman correlation analysis was performed to access the existence of a correlation between variables. Mann-Whitney-Wilcoxon Test or Student’s t test was used to evaluate the comparison between groups for continuous variables. Categorical variables were compared between groups using Chi-square or Fisher’s exact tests. All statistical analyses were performed in R software (version 3.6.2; https://www.r-project.org/) and they were 2-sided, with a P-value of less than 0.05 considered statistically significant.

## Results

### Association Between TP53 Mutation and Immunophenotype and Tumor Mutation Burden in Muscle-Invasive Bladder Cancer

**Figure 1:**
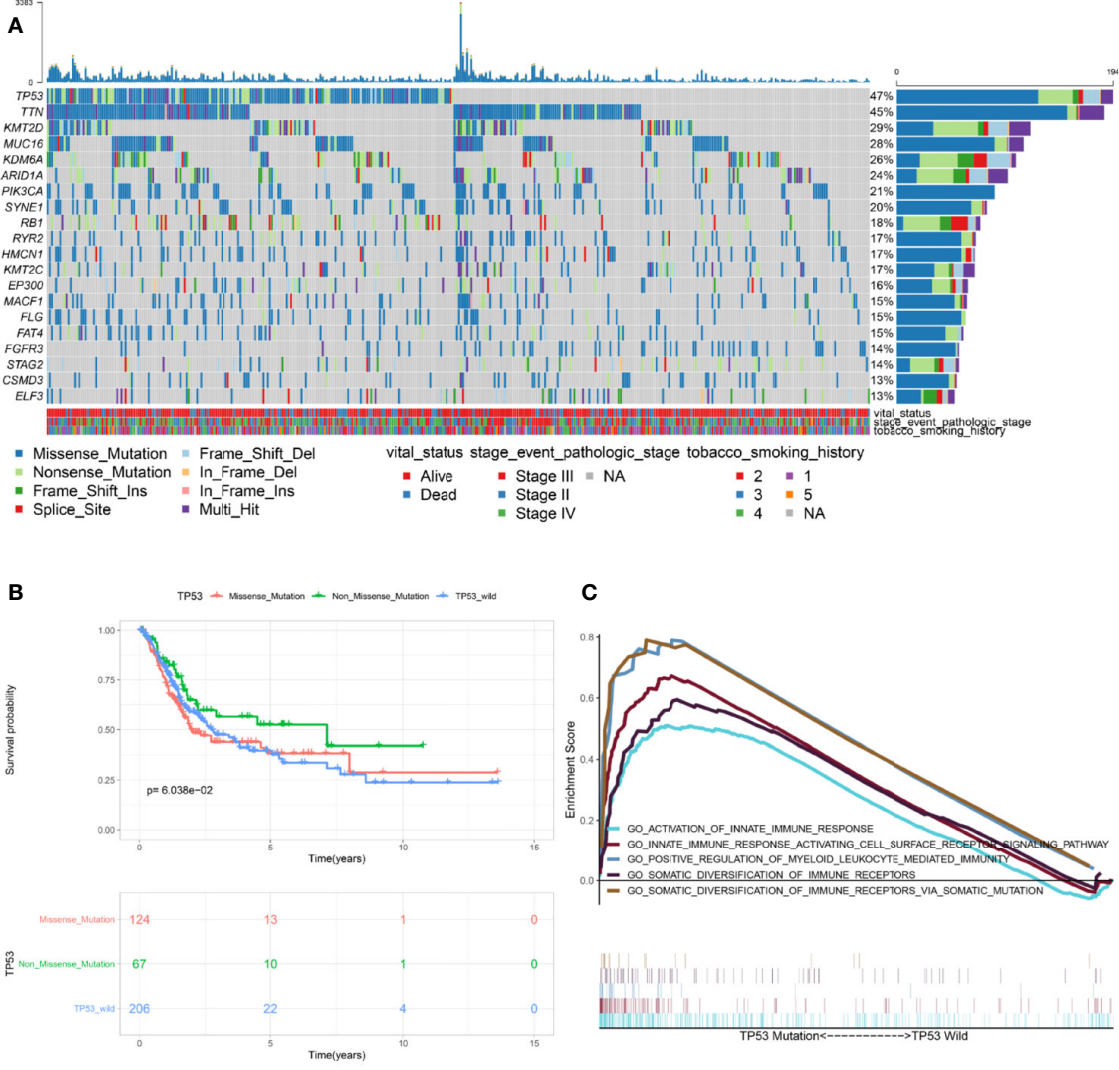
Association between TP53 mutation and immunophenotype in MIBC. **(A)** Overview of somatic mutations in all TCGA-MIBC samples. **(B)** Kaplan-Meier survival analysis of the different types of TP53 mutations. **(C)** Significant enrichment of immune-related biological processes in TP53^MUT^ MIBC patients compared with that in TP53^WT^ MIBC patients. (TCGA, The Cancer Genome Atlas; MIBC, muscle-invasive bladder cancer; MUT, mutation; WT, wild type.)

As shown in **Figure 1A**, TP53 mutation was the most frequent type in TCGA-MIBC cohort (47%), with missense mutation being the main type. In order to determine the prognosis of different types of TP53 mutation, we conducted stratification analysis based TP53 patterns which combined the nonsense mutation, frameshifts (deletions and insertions), in frame shift deletion, splice site and multi-hit mutations to non-missense mutations. The Kaplan-Meier survival analysis suggested that patients with missense mutations have a poorer prognosis when compared with non-missense mutations (**Figure 1B**). Previous studies found that TP53 mutation is associated with the pathological staging and prognosis of MIBC. However, research on the association between TP53 mutation and immunophenotype in MIBC has not matured. Therefore, for the first time, this study used RNA sequencing data and clinical information of TCGA-MIBC patients to identify immune-related biological processes related to TP53 status. GSEA analysis showed that TP53^mut^ MIBC patients were significantly enriched in 657 biological processes, out of these, five were immune-related biological processes (**Figure 1C**). In contrast, no immune-related biological processes were enriched in the TP53^wt^ MIBC patients. In addition, the proportion of TP53^mut^ MIBC patients was significantly high among high-TMB group compared with low-TMB group (Chi-square test, *P* value <0.001; **Figure S1A**). The TMB of TP53^mut^ MIBC patients was higher than TP53^wt^ TMB patients (Student’s t test, *P* value = 0.038; **Figure S1B**), suggesting that the TP53 mutation was closely associated with high TMB.

### Identification of Differentially Expressed Immune-Related Genes Between TP53^mut^ and TP53^wt^ Muscle-Invasive Bladder Cancer Patients

**Figure 2:**
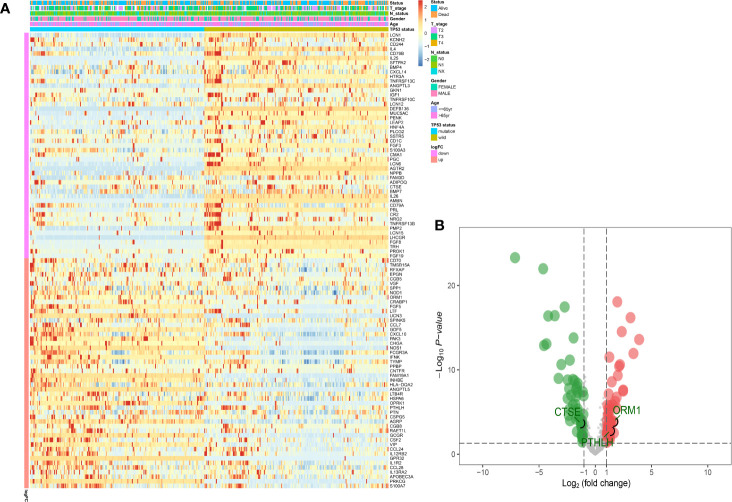
Identification of DEIGs between TP53^MUT^ and TP53^WT^ MIBC patients. The heatmap **(A)** and a volcano plot **(B)** were used to visualize the identified DEIGs. (DEIGs, differentially expressed immune-related genes; MUT, mutation; WT, wild type; MIBC, muscle-invasive bladder cancer.)

Based on the results obtained from the GSEA analysis, the TP53 mutation was closely related to immune-related biological processes, hence, MIBC patients were subdivided into TP53^mut^ and TP53^wt^ groups, then DEIGs were explored to further identify the correlations between the TP53 mutation and immunophenotype in MIBC. A total of 50 upregulated genes and 49 downregulated genes were identified (FDR < 0.05 and |log2- FC| > 1.0) (**Figures 2A, B**). The differentially expressed gene analysis using the “edgeR” package is shown in supplement **Table S1**. As shown in **Figure S2A**, significantly enriched BP of DEIGs were detected, including B cell proliferation, second messenger mediated signaling and humoral immune response. Several CC GO terms were detected, including external side of plasma membrane, secretory granule lumen and cytoplasmic vesicle lumen (**Figure S2B**). In GO terms of MF, receptor ligand activity, signaling receptor activator activity and cytokine activity were significantly enriched terms (**Figure S2C**). According to the KEGG pathway analysis, cytokine-cytokine receptor interaction, IL−17 signaling pathway and natural killer cell mediated cytotoxicity were mostly associated with the DEIGs (**Figure S2D**).

### Construction and Evaluation of TP53-Associated Immune Prognostic Signature in the The Cancer Genome Atlas Cohort

**Figure 3:**
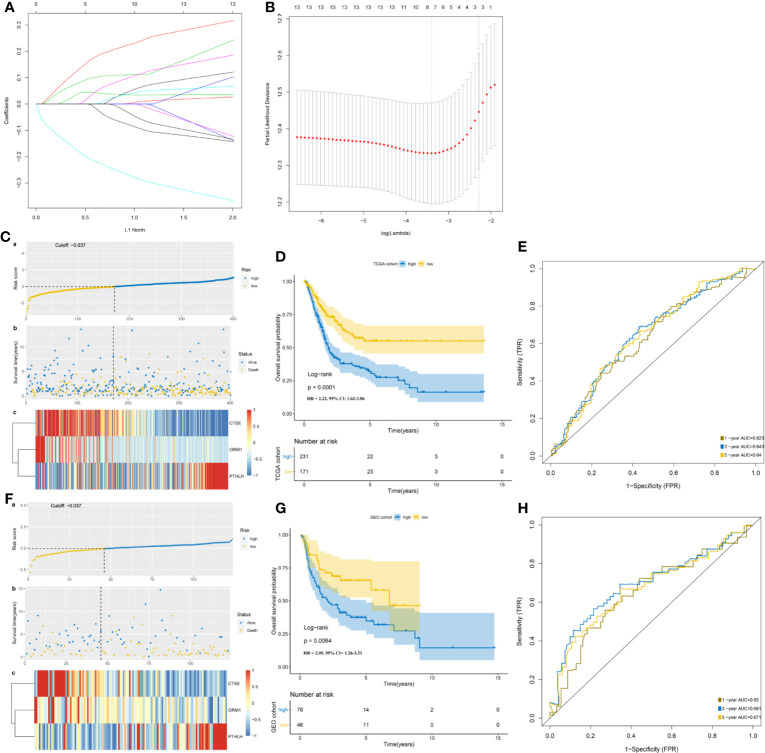
Development and validation of TP53-associated immune prognostic signature (TIPS) for MIBC. **(A)** LASSO coefficients profiles of 13 genes. **(B)** LASSO regression with 10-fold cross-validation obtained three prognostic genes that error is within one standard error of the minimum (lambda.1se). **(C)** The distribution of risk scores (a), survival status (b) and genes expression levels of MIBC patients (c) in the TCGA cohort. **(D)** Kaplan-Meier survival analysis of TIPS in the TCGA cohort. **(E)** Time-dependent ROC analysis of TIPS in the TCGA cohort. **(F)** The distribution of risk scores; (a) survival status; (b), and genes expression levels of MIBC patients; (c) in the GEO cohort. **(G)** Kaplan-Meier survival analysis of TIPS in the GEO cohort. **(H)** Time-dependent ROC analysis of TIPS in the GEO cohort. (TCGA, The Cancer Genome Atlas; GEO, Gene Expression Omnibus; MIBC, muscle-invasive bladder cancer).

Univariate Cox regression analysis was performed, where 13 of the 99 DEIGs were identified to be significantly associated with OS in TCGA-MIBC patients (**Table S2**). For further screen the 13 prognostic DEIGs, LASSO analysis was applied (**Figures 3A, B**). The LASSO analysis was used to shrink all regression coefficients toward zero and to select variables simultaneously; and the optimal lambda value were determined through 10-fold cross-validations. Finally, we identified TIPS using multivariate Cox regression analysis that was significantly associated with OS in MIBC patients. The risk score of each TCGA-MIBC patient was calculated as follows: [−0.42 × Expression value of ORM1] + [0.17 × Expression value of PTHLH] + [−0.39 × Expression value of CTSE] (**Table S3**). **Figure 2B** showed the differential expression of these three genes between TP53^mut^ and TP53^wt^ MIBC patients. Further, the TCGA-MIBC patients were divided into either low- or high-risk prognostic group based on −0.037 determined by X-tile 3.6.1 software (**Figures S3A, B**). The risk score distribution, gene expression, and OS status of TCGA-MIBC patients were shown and ranked based on risk score values of the TIPS (**Figure 3C**).

In addition, Kaplan-Meier survival analysis showed that high-risk prognostic group with the higher risk score had a significantly poorer prognosis than low-risk prognostic group with the low-risk score (HR = 2.22, 95% CI = 1.62–3.06, *P* < 0.0001) (**Figure 3D**). The AUCs for 1-, 3-, and 5-year OS predictions for TIPS were 0.625, 0.643, and 0.640 respectively, indicating that TIPS had a satisfactory sensitivity and specificity (**Figure 3E**). C-index of the TIPS was 0.62 (95%CI: 0.57–0.66, P<0.0001). As shown in **Figures S4A to C**, the AUCs for 1-, 3- and 5-year OS predictions of TIPS were higher than AUCs of age, gender, T stage, N status and TMB in the TCGA cohort.

### Validation and Evaluation of TP53-Associated Immune Prognostic Signature in the Gene Expression Omnibus Cohort

To evaluate the robustness of TIPS, its performance was assessed in the GEO cohort, which consisted of 124 MIBC patients. The patients in the GEO cohort were stratified into low- and high-risk prognostic groups using the same formula and the same cutoff value (−0.037) obtained from the TCGA cohort (**Table S4**). The risk score distribution, gene expression, and OS status of patients in the GEO cohort are shown in **Figure 3F**. In comparison with the high‐risk prognostic group in the GEO cohort, a significantly higher survival rates were observed in the low-risk prognostic group (HR = 2.05, 95% CI= 1.26–3.33, *P* = 0.0084) (**Figure 3G**). This was in line with the results of the TCGA cohort. Moreover, the TIPS achieved an AUC of 0.650 at 1 year, 0.691 at 3 years, and 0.671 at 5 years (**Figure 3H**). C-index of the TIPS was 0.61 (95%CI: 0.55–0.67, P<0.0001). The above results demonstrate the TIPS is effective in predicting the OS of MIBC patients.

### Gene Set Enrichment Analysis of the TP53-Associated Immune Prognostic Signature Genes

To further investigate enriched KEGG pathways of CTSE, ORM1 and PTHLH in MIBC, GSEA was performed based on TCGA-MIBC RNA-seq data. As shown in **Figure S5**, genes in the high expression groups of CTSE (**A**) were mainly enriched in the metabolism-related pathway, such as “alpha linolenic acid metabolism,” “arachidonic acid metabolism” and “drug metabolism cytochrome P450.” Meanwhile, the “Natural killer cell mediated cytotoxicity,” “complement and coagulation cascades,” and “cytokine-cytokine receptor interaction” were enriched in the high expression groups of ORM1 (**B**) and PTHLH (**C**), whereas “T cell receptor signaling pathway” and “Nod like receptor signaling pathway” were enriched in ORM1 and PTHLH high-expression groups, respectively. The results of GSEA revealed that ORM1 and PTHLH correlated with immune signaling pathways.

### Immune Landscape Between the Low- and High-risk Prognostic Groups

**Figure 4:**
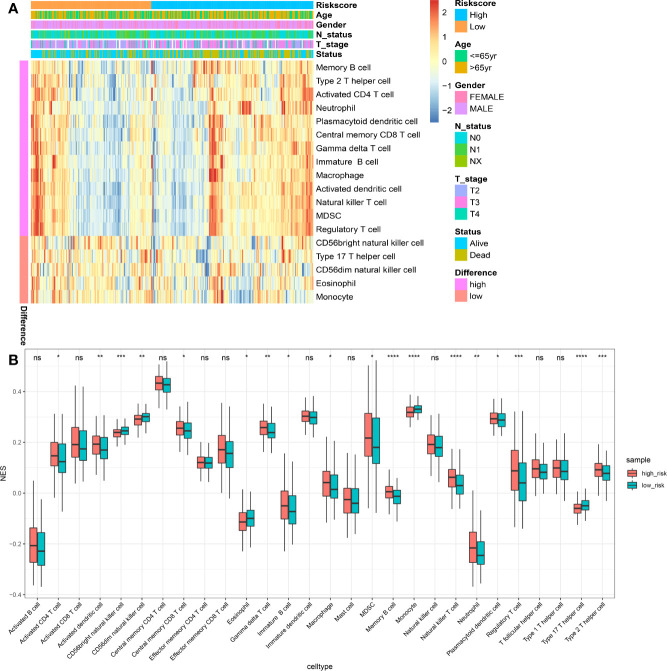
Immune landscape between the high- and low-risk MIBC patients. **(A)** The risk score, age, gender, T stage, N status and overall survival were used as patient annotations. Red represented high abundance and blue represented low abundance. **(B)** The abundance of each TIL in high- and low-risk MIBC patients. The asterisks indicated the statistical *P* value (*P < 0.05; **P < 0.01; ***P < 0.001; ****P < 0.0001). (TIL, tumor-infiltrating lymphocytes; MIBC, muscle-invasive bladder cancer). ns, no significance.

**Figure 5:**
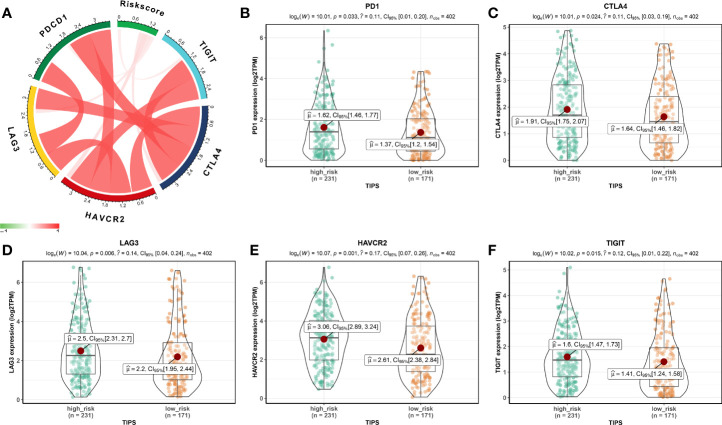
Association between TIPS and biomarkers for immunotherapy. **(A)** Correlation between risk score and the expression of PD1, CTLA4, LAG3, HAVCR2, and TIGIT. PD1 **(B)**, CTLA4 **(C)**, LAG3 **(D)**, HAVCR2 **(E)**, and TIGIT **(F)** gene expression differences between high- and low-risk MIBC patients. (TIPS, TP53-associated immune prognostic signature; MIBC, muscle-invasive bladder cancer).

Using the ssGSEA method, the differences in 28 subpopulations of TILs in the MIBC immune infiltrates between low- and high-risk prognostic groups were identified. **Figures 4A, B** are a summary of the results obtained from the 402 MIBC patients, showing that multiple subpopulations of TILs were significantly different between low- and high-risk prognostic groups. Among them, it was worth noting that the high-risk prognostic group had a significantly higher abundance of Tregs, TAM, and MDSCs as well as significantly lower abundance of CD56^bright^ NK cells compared to the low-risk prognostic group (*P* < 0.05). The heterogeneity of TILs in the MIBC immune infiltrates indicated that the TILs may be served potential prognostic biomarkers, immunotherapy targets, and might exhibit important clinical significance. Besides, it was found that the risk score was positively related to the expression of critical immune checkpoints in the TCGA cohort, including PD1, CTLA4, LAG3, HAVCR2, and TIGIT (*P*<0.05) (**Figure 5A**). Also, the differential expression of PD1, CTLA4, LAG3, HAVCR2, and TIGIT between the low- and high-risk prognostic groups were analyzed. Results showed that the expression of PD1, CTLA4, LAG3, HAVCR2, and TIGIT was significantly higher in high-risk prognostic group than in low-risk prognostic group (**Figures 5B–F**), implying that the bad prognosis of high-risk prognostic group might be associated with the immunosuppressive microenvironment.

### Association Between TP53-Associated Immune Prognostic Signature and APOBEC-signature Mutation

The high APOBEC-signature mutation load may stimulate a natural host immune reaction to curb tumor growth and metastasis and was associated with better prognosis of MIBC (36). The expression of APOBEC3A and APOBEC3B positively correlated with levels of APOBEC-signature mutagenesis, which was ubiquitous and carcinogenic (36, 37). The file including the information of the APOBEC-signature mutation load was downloaded from the study of Robertson et al. (https://www.ncbi.nlm.nih.gov/pmc/articles/PMC5687509/#) to analyze the association between TIPS and APOBEC-signature mutation. Low-risk prognostic group had a high APOBEC-signature mutation load and low expression of APOBEC3A and APOBEC3B, but this was not statistically significant (**Figures S6A–C**).

### Chemotherapeutic and Immunotherapeutic Responses of Low- and High-risk Prognostic Groups

**Figure 6:**
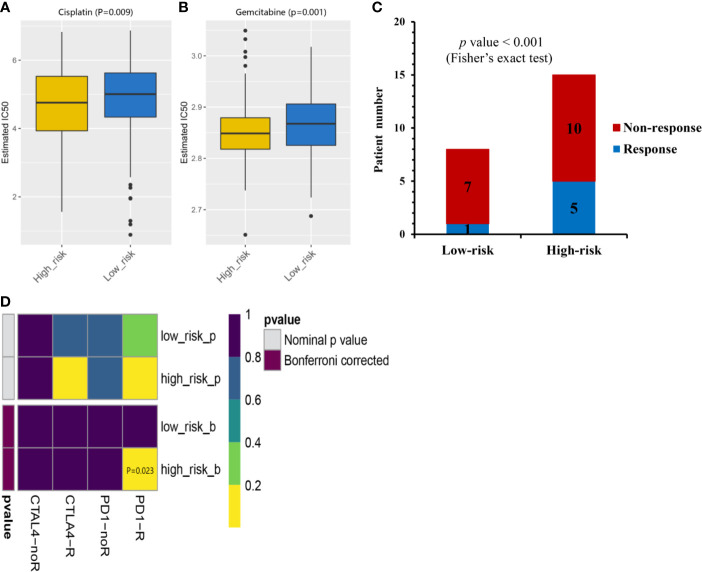
Chemotherapeutic and immunotherapeutic responses of high- and low-risk MIBC patients. The box plots of the estimated IC50 for cisplatin **(A)** and gemcitabine **(B)** indicated differential chemotherapeutic response between the high- and low-risk MIBC patients. **(C)** The proportion of patients with response to NAC in low- or high-risk prognostic groups in GSE52219 data set. Response, blue; Non-response, red. **(D)** Immunotherapeutic responses to anti-PD-1and anti-CTLA-4 treatments of high- and low-risk MIBC patients. CR/PR, blue; SD/PD, red. (NAC, neoadjuvant chemotherapy; MIBC, muscle-invasive bladder cancer).

Given that chemotherapy is a common approach in the treatment of MIBC, this study attempted to evaluate the response of low- and high-risk prognostic groups to the treatment by gemcitabine and cisplatin. The IC50 of each TCGA-MIBC patient was estimated and as shown in **Figures 6A, B**, it resulted in a significant difference in the estimated IC50 between low- and high-risk prognostic groups for the two chemotherapy drugs, where the high-risk prognostic group was more sensitive to the treatment (P = 0.009 for cisplatin, P = 0.001 for gemcitabine). As shown in **Figure 6C**, the proportion of NAC responders was significantly high among high-risk prognostic group compared with the low-risk prognostic group in GSE52219 data set (Fisher’s exact test, *p* value < 0.001). High-risk prognostic group were more sensitive to NAC and may benefit from it which was consistent with the above predictions. To determine the potential of the risk score as a biomarker for immunotherapy, the difference in the estimated immunotherapeutic response of low- and high-risk prognostic groups was identified. Interestingly, the analysis suggested that high-risk prognostic group might be more responsive to anti–PD-1 therapy (Bonferroni corrected *P* = 0.023) (**Figure 6D**).

### The TP53-Associated Immune Prognostic Signature Is Independent of Clinical Characteristics

**Figure 7:**
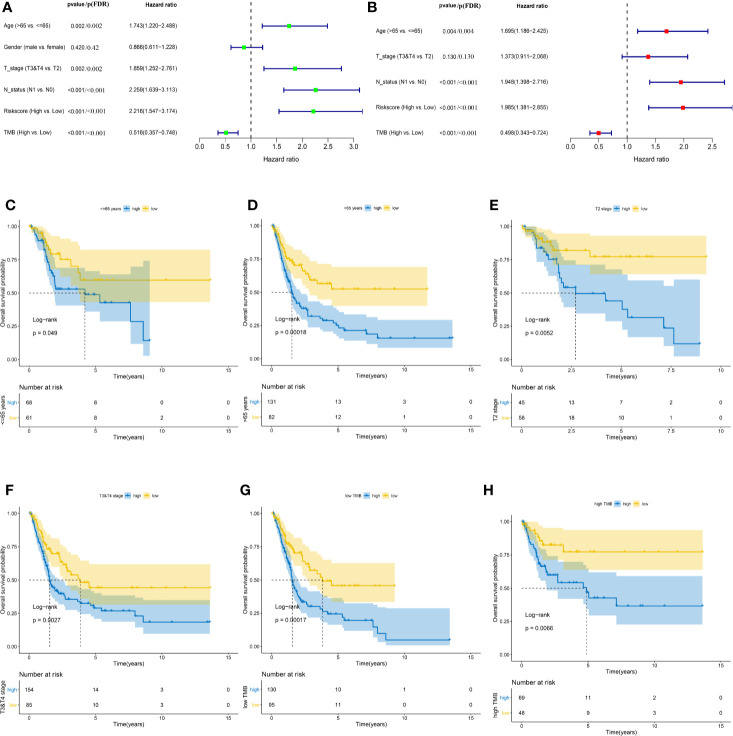
Identifying the independent clinical characters. Forrest plot of univariate **(A)** and multivariate **(B)** Cox regression analysis in the TCGA cohort. Kaplan-Meier survival analysis of TIPS in different subgroups including <=65 years **(C)** >65 years **(D)** T2 **(E)** T3&T4 **(F)** low TMB **(G)** and high TMB **(H)** subgroups. (FDR, false discovery rate; TCGA, The Cancer Genome Atlas; MIBC, muscle-invasive bladder cancer; TMB: tumor mutation burden).

Baseline characteristics of 342 MIBC patients in the TCGA cohort are summarized in **Table 1**. TMB was stratified into low and high TMB groups according to 5.55/Mb determined by X-tile 3.6.1 software (**Figure S7**). Regarding the Schoenfeld Individual Test, the *P* value of five clinical characters, including age (A), gender (B), T, stage (C), N status (D), TIPS (E) and TMB (F) was >0.05 and the PH assumption could not be considered violated (**Figure S8**). Univariate and multivariate Cox regression analyses were performed to explore the independence of TIPS to clinical characteristics in the TCGA cohort. After adjustment for clinical characteristics and multiple testing using FDR method, the TIPS remained an independent prognostic signature indicating its robustness in independently predicting the OS of MIBC patients (**Figures 7A, B**). Subsequently, Kaplan-Meier survival analysis was used to analyze the predictive value of the TIPS in different subgroups stratified by age, T stage and TMB, showing that the high-risk prognostic group had a poorer OS compared to low-risk prognostic group in ≤65 years, >65 years, T2, T3&T4, low and high TMB subgroups (**Figures 7C–H**). We further analyzed the relationship between the risk score and the clinical characteristics of MIBC (including age, T stage and N status). In terms of T stage, T3&T4 stage patients had higher risk scores than T2 stage patients in the TCGA data set but the risk scores for the N1 status and >65 years patients were not higher than those for the N0 status and ≤65 years patients (**Figures S9A–C**).

**Table 1:** Baseline characteristics of 342 MIBC patients in the TCGA cohort.

Characteristic	TCGA cohort
Patients (n)	342
mean follow-up time (months, range)	26.8 (1.0–165.9)
Age (years)	
≤65	129 (37.6)
>65	213(62.4)
Gender, n (%)	
female	91 (36.8)
male	251 (73.2)
AJCC pathologic T stage, n (%)	
T2	103 (30.0)
T3 and T4	239(70.0)
AJCC pathologic N status, n (%)	
N0	219 (64.1)
N1	123 (35.9)
Risk score, n (%)	
High	199 (58.6)
Low	143 (41.4)
Survival status, n (%)	
Dead	151 (44.3)
Alive	191 (55.7)
Tumor mutation burden, n (%)	
High	117 (34.2)
Low	225 (65.8)

### Development and Evaluation of the Nomogram Based on the TP53-Associated Immune Prognostic Signature

**Figure 8:**
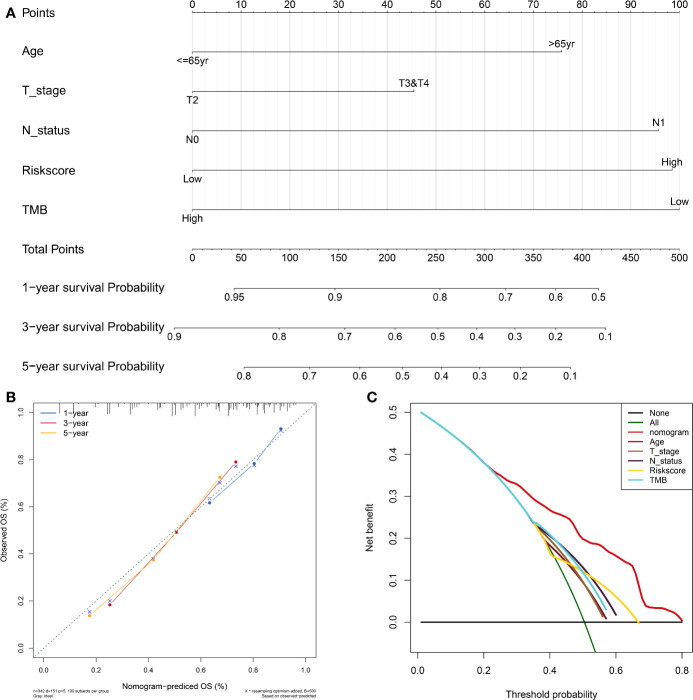
Development and evaluation of the nomogram based on the TIPS. **(A)** Nomogram integrated the risk score, age, T stage, N status and TMB to predict 1-, 3-, and 5-year OS of MIBC. **(B)** The calibration plot for internal validation of the nomogram. **(C)** DCA for 5-year OS prediction shows that the nomogram has the highest net benefit across 0% to 80% threshold probabilities. (DCA, decision curve analysis; TCGA, The Cancer Genome Atlas; MIBC, muscle-invasive bladder cancer; TIPS, TP53-associated immune prognostic signature; TMB: tumor mutation burden).

To facilitate the clinical decision making, a nomogram was developed based on the TIPS to predict 1-, 3-, and 5-year OS of the TCGA-MIBC patients. The risk score, age, T stage, N status and TMB were selected into the nomogram by a stepwise Cox regression model (**Figure 8A**). And as shown in **Figure S4A-C**, the AUC for 1-, 3- and 5-year OS predictions of the nomogram was higher than AUC of the TIPS, age, gender T stage, N status and TMB in the TCGA cohort. The C-index of the nomogram was 0.69 (95% CI, 0.64–0.73), while that of the risk score was 0.62 (95% CI, 0.57–0.66) in the TCGA cohort and 0.61 (95% CI, 0.55–0.67) in the GEO cohort. The calibration plots showed consistency in predicting OS of the nomogram with the actual probability of OS at 1-, 3- and 5-year (**Figure 8B**). Moreover, the DCA for 5-year OS prediction showed that the nomogram had the highest net benefit across 0% to 80% threshold probabilities (**Figure 8C**). Meanwhile, the net benefit of the TIPS was higher than age, T stage, N status and TMB.

## Discussion

MIBCs are primarily characterized by their rapid progression, high metastatic potentials, and poor prognosis (38). Evidence from recent literature revealed that a high abundance of NK cells, T cells, and B cells correlate with an inflammatory response targeting MIBC whereas the abundance and spatial distribution of TILs are associated with a favorable prognosis of MIBC patients after standard treatment (39). TP53 gene is the most common mutation types in MIBC (17). BC patients with TP53 mutation showed a poorer prognosis and higher grade of pathology compared to patients without TP53 mutation (18, 40). In addition, TP53 mutation has been confirmed as an indicator of anti-PD1 therapy in lung cancer (41) and can be associated with upregulated interferon-gamma levels and expression of immune checkpoints, as well as activation of effector T cells in lung adenocarcinoma (21). Although, Stadler et al. (42) confirmed that neither the prognostic value of p53 nor the benefit of combination methotrexate, vinblastine, doxorubicin, and cisplatin (MVAC) adjuvant chemotherapy in patients with p53-positive tumors, whereas this study exist some limitations such as the number of patients randomly assigned and, more importantly, who received treatment as assigned, was much lower than anticipated. In addition, the mechanism of TP53 mutation in regulating TME and prognosis of MIBC is unclear. Hence, it is vital to explore the role of the TP53 mutation in regulating TME and further reveal the relationship between the prognosis of MIBC and the TP53 mutation.

In this study, DEIGs were identified in MIBC patients with the TP53 mutation, and novel prognostic signatures and therapeutic targets were provided for the management of MIBC. GSEA based on TP53 status revealed that GO-BP terms of TP53^mut^ MIBC patients were significantly associated with immune-related biological processes. The TIPS was established based on three genes (ORM1, PTHLH, and CTSE) to identify high-risk prognostic group with a poorer OS and having more potential to respond to anti-PD1, gemcitabine and cisplatin therapies. Furthermore, we successfully validated the feasibility of the TIPS to predict chemotherapeutic benefit in the available observed data. Previous analysis suggested that p53 alteration was not a suitable prognostic or predictive biomarker (42) due to the limitation of the power of a single biomarker and lack of comprehensive p53 pathway analysis. The current data promises to fill these gaps, combined TP53 mutation with TIPS may be useful to predictive benefit from NAC.

Functional enrichment analyses were conducted to identify the potential molecular mechanisms of the three TIPS genes (ORM1, PTHLH, and CTSE). As expected, the results revealed that ORM1 and PTHLH were associated with tumor immunity, such as antigen processing and presentation, T cell receptor signaling pathway and cytokine-cytokine receptor interactions. Nevertheless, the CTSE was related to lipid metabolism. ORM1 plays an important role in modulating the activity of the immune system during the acute-phase reaction. Fan et al. (43) reported that ORM1 and other acute reactants may function as blocking factors to protect tumor cells against immunological attack, thus contributing to the "immune escape" of the tumor. Moreover, elevated urine ORM1 was positively correlated with the clinicopathological parameters of BC, which indicated ORM1 as a potential biomarker in BC (44). PTHLH is a member of the parathyroid hormone family, which is responsible for most cases of humoral hypercalcemia of malignancy (45). PTHLH functions as a critical regulator of cellular and organ growth, development, migration, survival and of epithelial calcium ion transport (45). Recently, Chen et al. revealed that silencing of transforming growth factor-β-activated kinase 1 (TAK1) in BC cells promote the development of cancer cells by upregulating PTHLH (46). CTSE is involved in antigen processing and the maturation of secretory proteins, which can regulate the processing of antigenic peptides during MHC class II-mediated antigen presentation. In RNA sequencing analysis, CTSE expression in BC organs was higher than that in normal bladder tissues (47). In addition, the expression of CTSE was significantly correlated with the progression to stage T2 to T4 BC (48). Taken together, previous investigations and our results suggest that the three genes (ORM1, PTHLH, and CTSE) may act as potential biomarkers and therapeutic targets for MIBC.

BC promotes the formation of a highly immunosuppressive microenvironment through various mechanisms geared toward preventing the production of effective anti-tumor immune response (49). Mechanisms of immune evasion in BC mainly include elevated immunosuppressive cells (e.g., Tregs, TAM, and MDSC) (49) and high expression of immune checkpoints (e.g., CTLA-4 and PD-1) (50). Notably, PD1 (Programmed cell death protein 1) is highly expressed on Treg cells of many cancers, suppressing the effector function of T cells, thus causing its exhaustion (51–53). Therefore, we speculate that low- and high-risk prognostic groups would exhibit a unique immune landscape and immunotherapeutic responses. Generally, our results demonstrated that a higher abundance of Tregs, TAM, and MDSCs and a lower abundance of CD56^bright^ NK cells was observed in high-risk prognostic group than low-risk prognostic group. The high abundance of CD56bright NK cells was associated with improved survival of MIBC patients (54). Interestingly, our study suggested that high-risk prognostic group showed high expression of PD1, CTLA4, LAG3, HAVCR2, and TIGIT and were more sensitive to anti-PD1 treatment. Being one of the most used chemotherapy regimens for BC (55), this study demonstrated that high-risk prognostic group were more sensitive to gemcitabine and cisplatin than low-risk prognostic group. The above findings revealed that the poor prognosis of high-risk prognostic group might be linked to a high degree of immunosuppression and low immunoreactivity in TME, thereby promoting tumor recurrence and metastasis. As a consequence, high-risk prognostic group might benefit from immunotherapy and chemotherapy.

It was demonstrated that risk score, age, pathological T stage, N status and TMB were significantly associated with OS of TCGA-MIBC patients, and further established TIPS as an independent prognostic factor of OS for MIBC. Additionally, age, pathological T stage, N status and TMB are important prognostic determinants for MIBC. However, prognosis differ among patients with similar clinical characteristics, implying that conventional clinical characteristics are insufficient to precisely predict prognosis. As a result, it is vital to explore more biomarkers that serve as prognostic signature and therapeutic targets. Moreover, our study is the first to identify TP53-associated immune prognostic signature for MIBC. TIPS provide a novel method to predict prognosis and offer guidance for therapeutic decisions of MIBC. TIPS can even predict the prognosis of MIBC patients with different subgroups stratified by clinical characteristics. Moreover, we constructed a nomogram combining TIPS with clinical characteristics to effectively predict prognosis. The nomogram demonstrated that TIPS is an effective signature in predicting OS of MIBC patients.

Our study has several limitations. This study was a retrospective design and existed heterogeneity due to comparisons between patients from different cohorts. Hence, the validation of the prospective cohort is even more necessary in this study. Nonetheless, our study showed that the benefits from NAC and immunotherapy differed between low- and high-risk prognostic groups.

In summary, we developed and validated TIPS based on three genes (ORM1, PTHLH, and CTSE) that exhibited an independent prognostic significance for MIBC patients. Further, a nomogram was constructed combining TIPS, age, pathological T stage, N status and TMB to accurately identify high-risk prognostic group. We found that high-risk prognostic group might benefit from NAC and anti-PD1 therapy. The use of TIPS could help clinicians make advanced personalized treatment decisions. Notably, our study provides a foundation for researchers to explore novel treatment strategies of MIBC. Nonetheless, clinical trials involving a larger cohort with longer follow-up are essential to validate our findings. Further insights regarding the functional role of ORM1, PTHLH, and CTSE in carcinogenesis might offer fundamental approaches in the treatment of MIBC.

## Data Availability Statement

Publicly available datasets were analyzed in this study. This data can be found here: TCGA database (https://cancergenome.nih.gov/) and GEO database (https://www.ncbi.nlm.nih.gov/geo/).

## Author Contributions

Data analyses were performed by XW and DL. CC, ZZ, MW, and WC assisted in collecting data. Funding was obtained by YL and DL. The manuscript was written by XW and DL, and was commented and revised by YL, CC, and ZZ. All authors participated in preparing the manuscript and approved the final submitted and published version. YL supervised the study. All authors contributed to the article and approved the submitted version.

## Funding

This study was supported by grants from the National Natural Science Foundation of China (81600542 and 81670643), the Natural Science Foundation of Guangdong Province (2020A1515010464), the Guangdong Basic and Applied Basic Research Foundation (grant 2019A1515110033), the Distinguished Young Talents in Higher Education Foundation of Guangdong Province (grant 2019KQNCX115 and 2020KZDZX1168), the China Postdoctoral Science Foundation (grant 2019M662865), and the Achievement cultivation and clinical transformation application cultivation projects of the First Affiliated Hospital of Guangzhou Medical University (grant ZH201908).

## Conflict of Interest

The authors declare that the research was conducted in the absence of any commercial or financial relationships that could be construed as a potential conflict of interest.

## Supplementary Material

Supplementary Figure 1 Association between TP53 mutation and TMB. **(A)** The proportion of patients with and without TP53 mutations in low- or high- TMB patients in the TCGA cohort. **(B)** Differences of risk score between high- and low- TMB patients. (TMB, tumor mutation burden; TCGA, The Cancer Genome Atlas).

Supplementary Figure 2 GO and KEGG analysis of DEIGs. **(A)** Chord plot shows the relationship between DEIGs and GO terms of biological process. **(B)** Chord plot shows the relationship between DEIGs and GO terms of cellular component. **(C)** Chord plot shows the relationship between DEIGs and GO terms of molecular function. **(D)** Chord plot shows the relationship between DEIGs and KEGG pathways. (GO, Gene Ontology; KEGG, Kyoto Encyclopedia of Genes and Genomes; DEIGs, differentially expressed immune-related genes).

Supplementary Figure 3 A, B. Estimation of the best cut off value for the risk score stratification as determined by the X-tile software.

Supplementary Figure 4 Comparison of the predictive power among nomogram, risk score, age, gender, T stage, N status and TMB in the TCGA cohort. 1- **(A)**, 3- **(B)**, and 5-year **(C)** time-dependent ROC analysis of nomogram, risk score, age, gender, T stage, N status and TMB. (TCGA, The Cancer Genome Atlas; TMB: tumor mutation burden).

Supplementary Figure 5 GSEA of KEGG pathway gene sets in CTSE **(A)**, ORM1 **(B)** and PTHLH (C). High expression versus low expression samples from TCGA database. Normalized enrichment score (NES) is shown in each plot. (GSEA, Gene set enrichment analysis; KEGG, Kyoto Encyclopedia of Genes and Genomes).

Supplementary Figure 6 Association between TIPS and APOBEC-signature mutation. **(A)** Differences of APOBEC-signature mutation load between high- and low- MIBC patients. **(B-C)** Differences expression of APOBEC3A **(B)** and APOBEC3B **(C)** between high- and low- MIBC patients. (MIBC, muscle-invasive bladder cancer; TIPS, TP53-associated immune prognostic signature).

Supplementary Figure 7
**(A, B)** Estimation of the best cut off value for TMB stratification as determined by the X-tile software. (TMB: tumor mutation burden).

Supplementary Figure 8 The Schoenfeld Individual Test of six clinical characters, including age **(A)**, gender **(B)**, T, stage **(C)**, N status **(D)**, TIPS **(E)** and TMB **(F)**. (TIPS, TP53-associated immune prognostic signature; TMB: tumor mutation burden).

Supplementary Figure 9 Show the distribution of the risk score in different T stage **(A)**, N status **(B)** and age **(C)** in TCGA cohort. (TCGA, The Cancer Genome Atlas).
