# Supplementary figures and images for: A TP53-Associated Immune Prognostic Signature for the Prediction of Overall Survival and Therapeutic Responses in Muscle-Invasive Bladder Cancer
Source: Front Immunol. 2020 Dec 17;11:590618. doi: 10.3389/fimmu.2020.590618 (PMC7774015; doi:10.3389/fimmu.2020.590618)

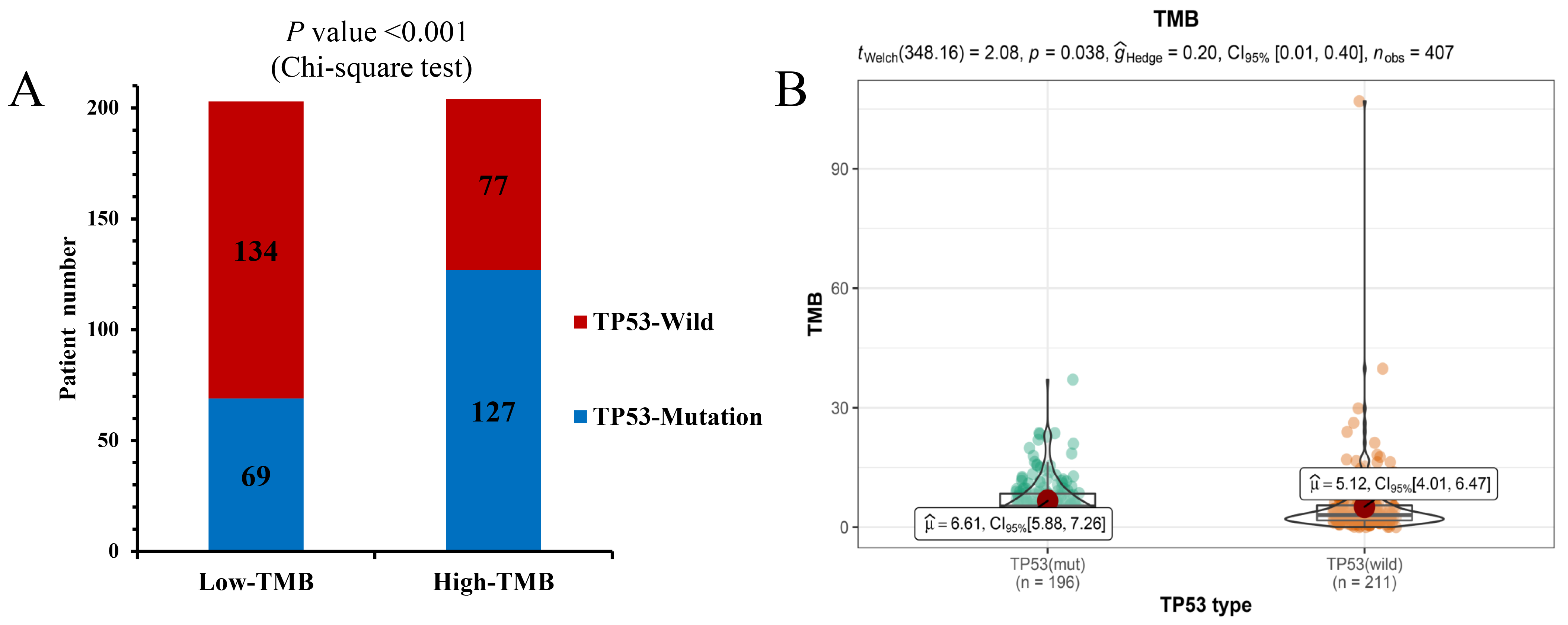

Supplement: Supplementary Figure 1 — Association between TP53 mutation and TMB. (A) The proportion of patients with and without TP53 mutations in low- or high- TMB patients in the TCGA cohort. (B) Differences of risk score between high- and low- TMB patients. (TMB, tumor mutation burden; TCGA, The Cancer Genome Atlas). [file Image_1.tif]

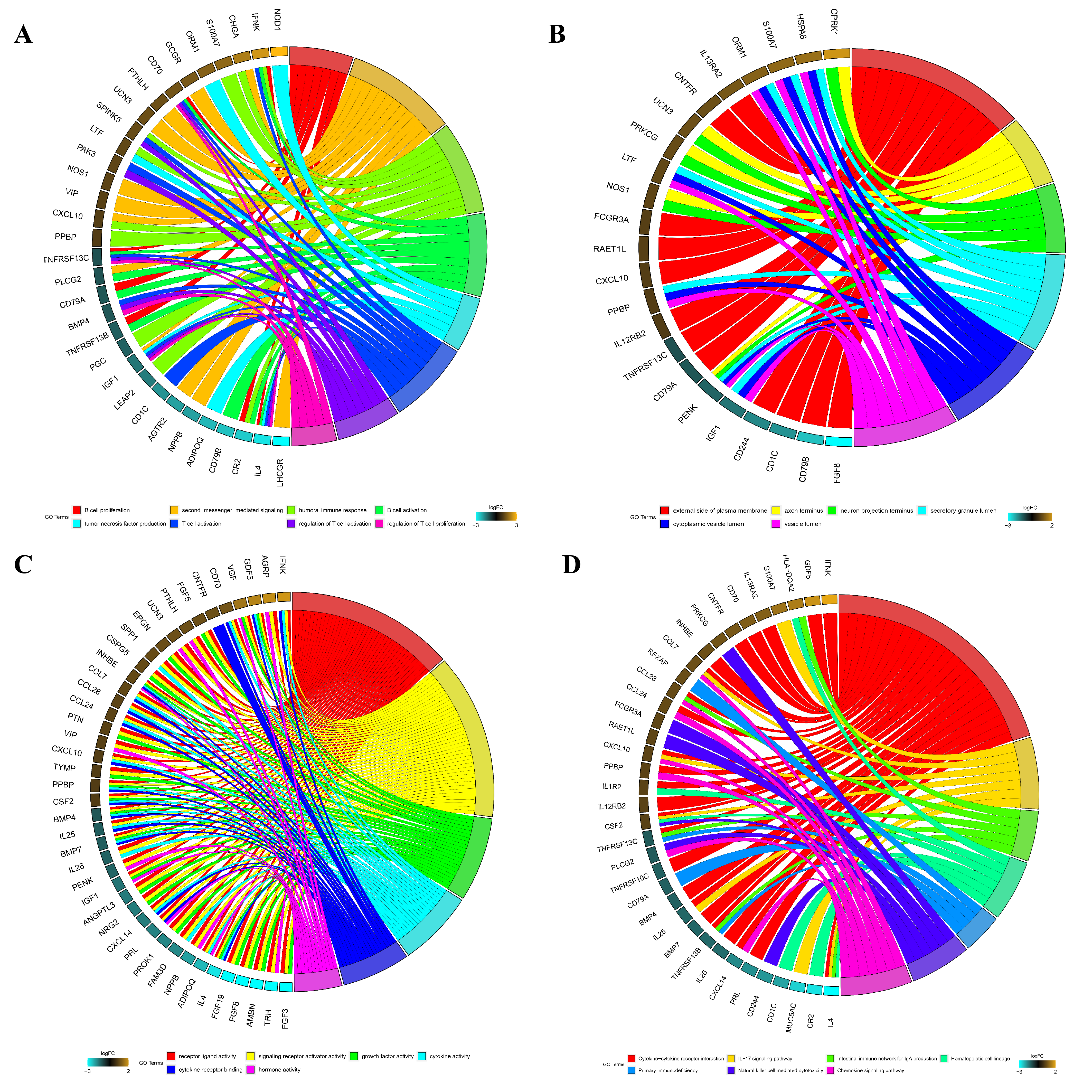

Supplement: Supplementary Figure 2 — GO and KEGG analysis of DEIGs. (A) Chord plot shows the relationship between DEIGs and GO terms of biological process. (B) Chord plot shows the relationship between DEIGs and GO terms of cellular component. (C) Chord plot shows the relationship between DEIGs and GO terms of molecular function. (D) Chord plot shows the relationship between DEIGs and KEGG pathways. (GO, Gene Ontology; KEGG, Kyoto Encyclopedia of Genes and Genomes; DEIGs, differentially expressed immune-related genes). [file Image_2.tif]

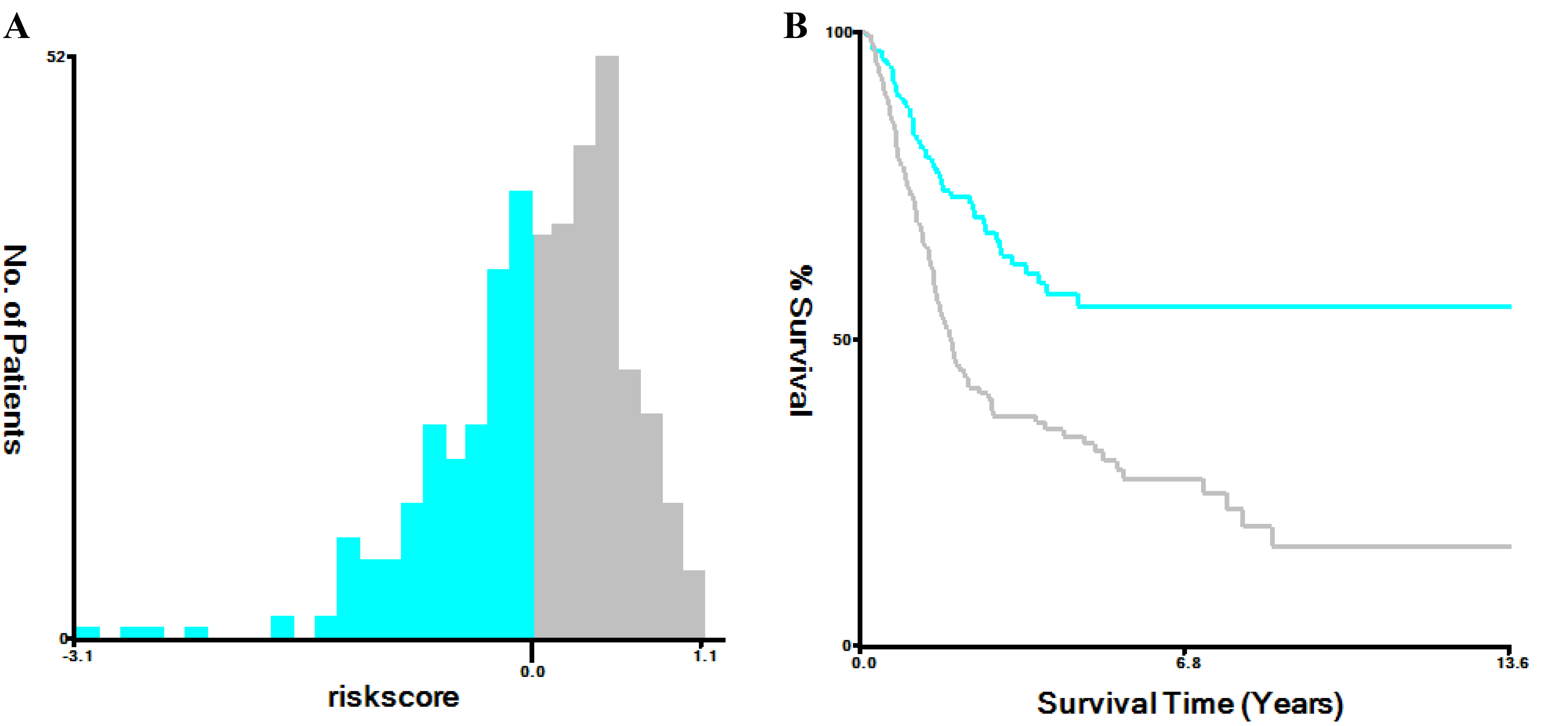

Supplement: Supplementary Figure 3 — A, B. Estimation of the best cut off value for the risk score stratification as determined by the X-tile software. [file Image_3.tif]

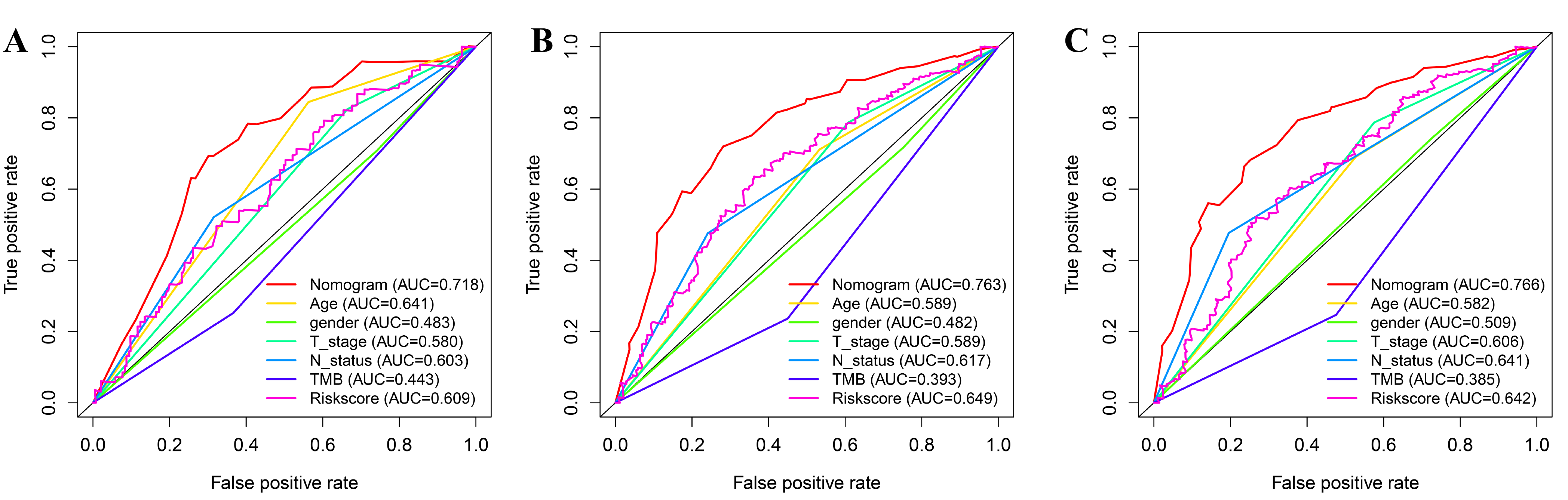

Supplement: Supplementary Figure 4 — Comparison of the predictive power among nomogram, risk score, age, gender, T stage, N status and TMB in the TCGA cohort. 1- (A), 3- (B), and 5-year (C) time-dependent ROC analysis of nomogram, risk score, age, gender, T stage, N status and TMB. (TCGA, The Cancer Genome Atlas; TMB: tumor mutation burden). [file Image_4.tif]

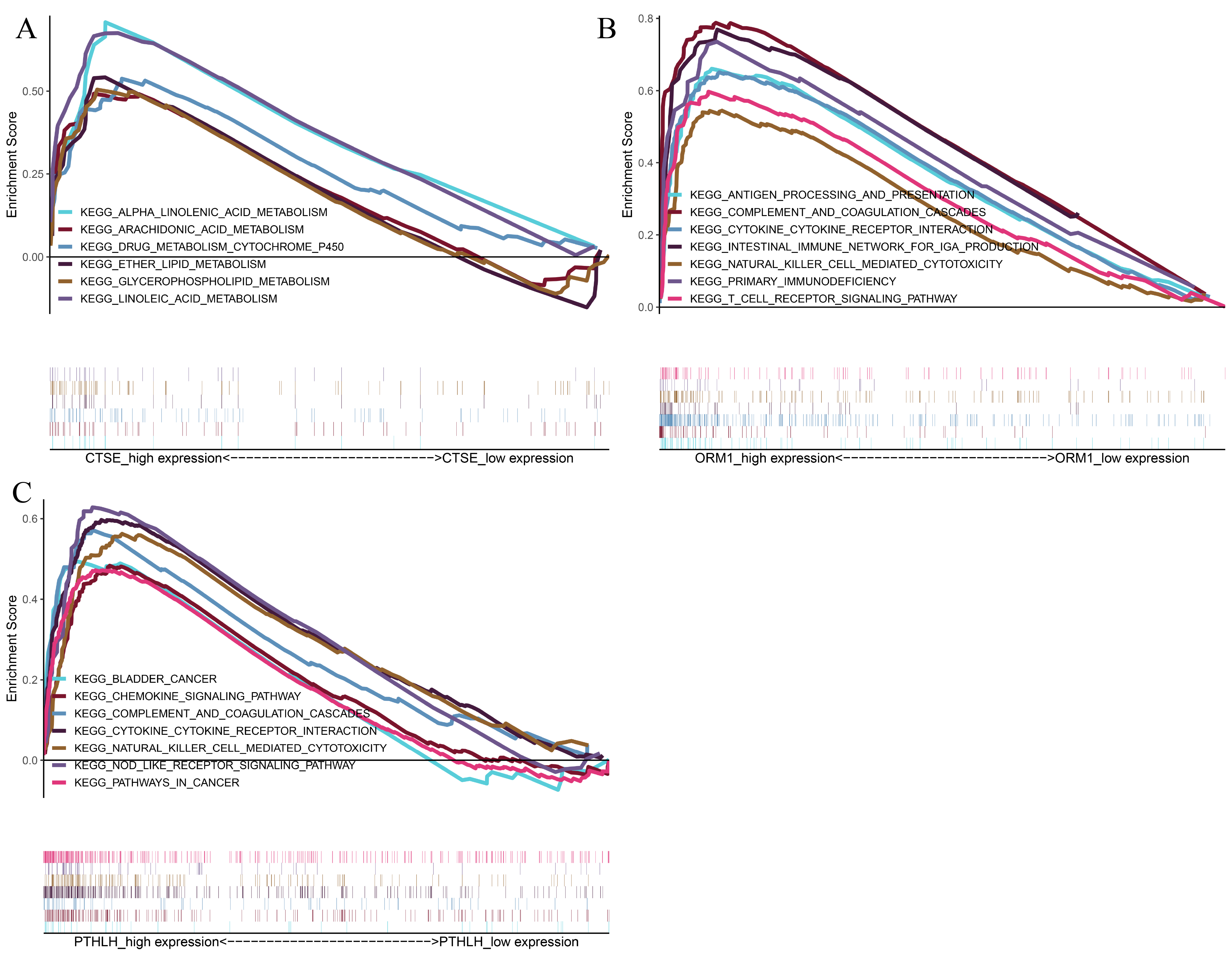

Supplement: Supplementary Figure 5 — GSEA of KEGG pathway gene sets in CTSE (A), ORM1 (B) and PTHLH (C). High expression versus low expression samples from TCGA database. Normalized enrichment score (NES) is shown in each plot. (GSEA, Gene set enrichment analysis; KEGG, Kyoto Encyclopedia of Genes and Genomes). [file Image_5.tif]

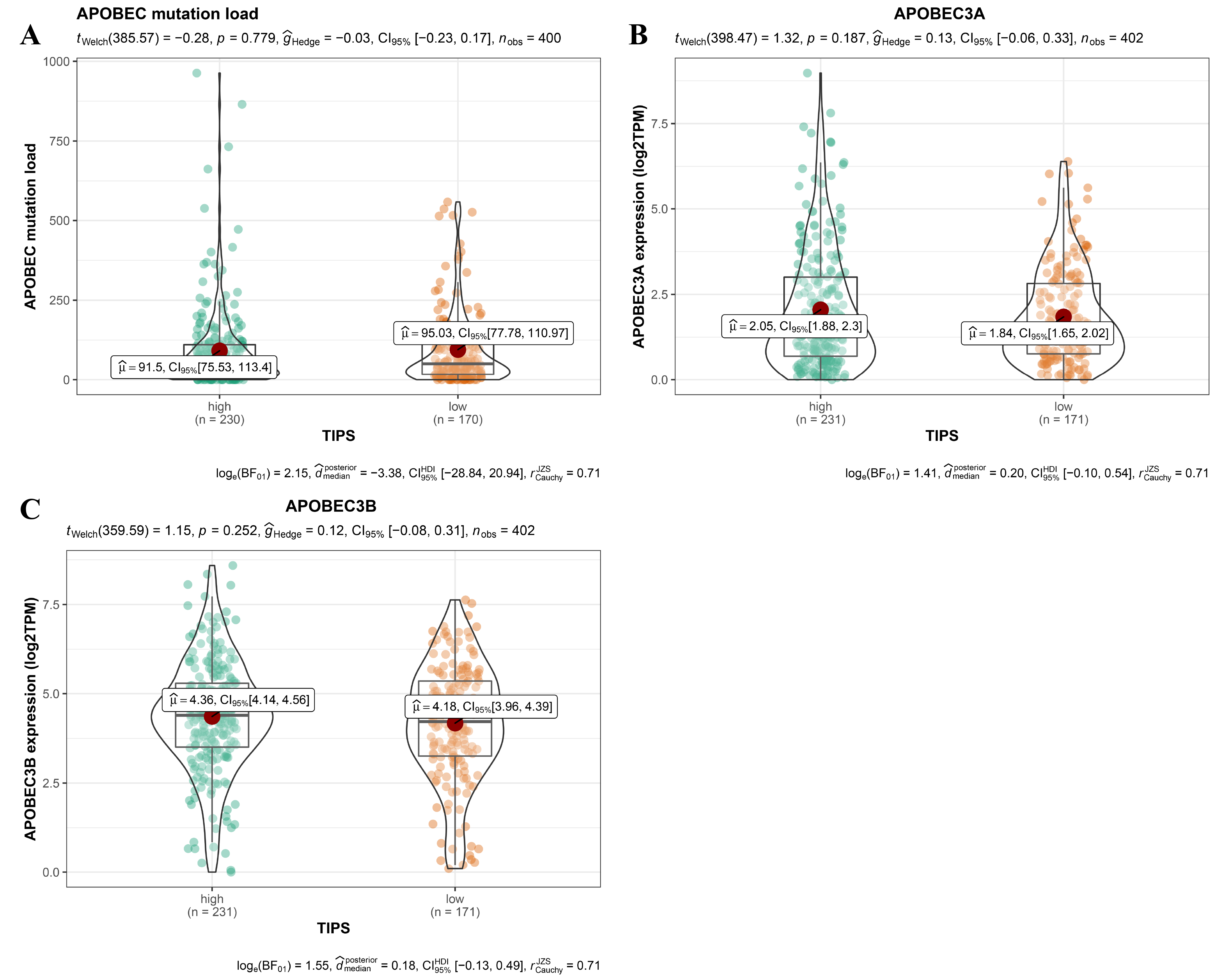

Supplement: Supplementary Figure 6 — Association between TIPS and APOBEC-signature mutation. (A) Differences of APOBEC-signature mutation load between high- and low- MIBC patients. (B-C) Differences expression of APOBEC3A (B) and APOBEC3B (C) between high- and low- MIBC patients. (MIBC, muscle-invasive bladder cancer; TIPS, TP53-associated immune prognostic signature). [file Image_6.tif]

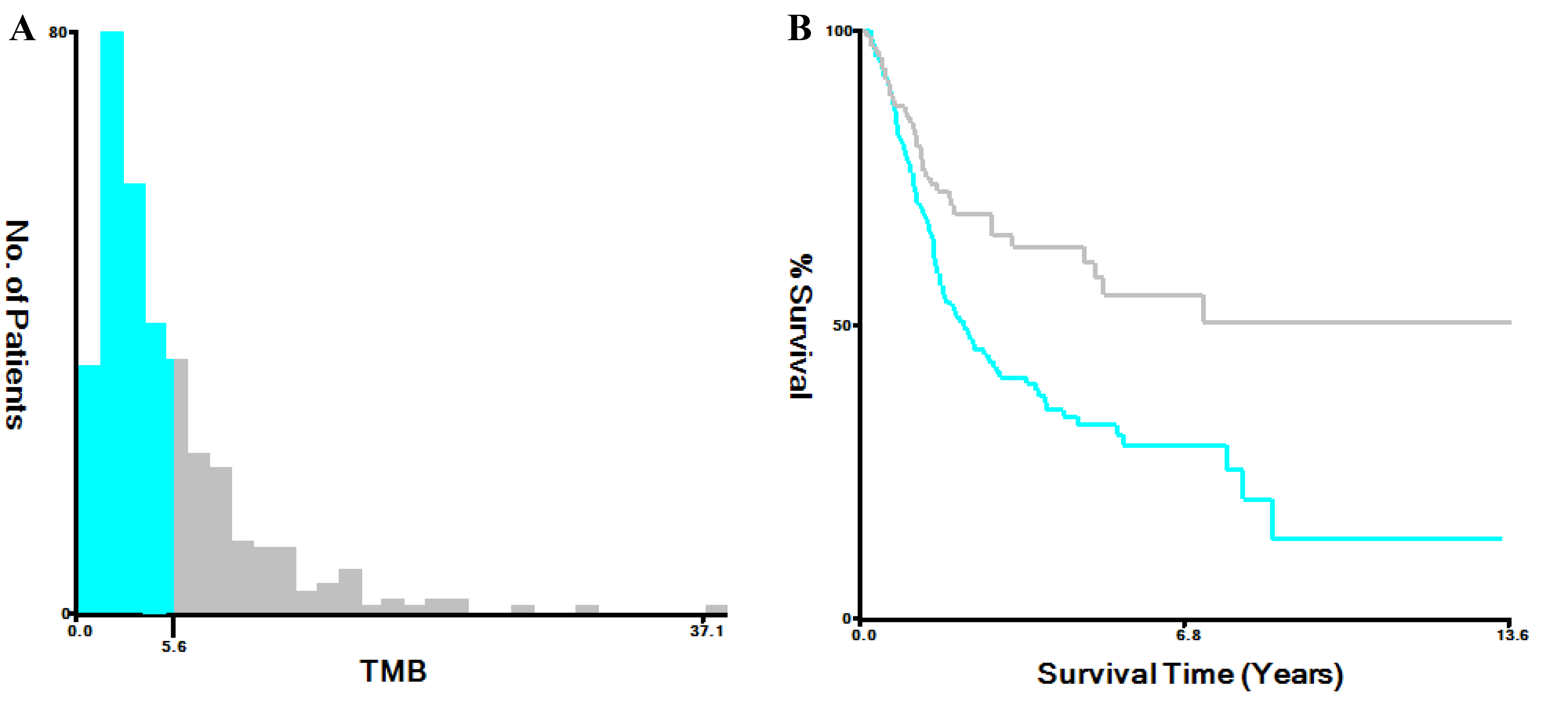

Supplement: Supplementary Figure 7 — (A, B) Estimation of the best cut off value for TMB stratification as determined by the X-tile software. (TMB: tumor mutation burden). [file Image_7.tif]

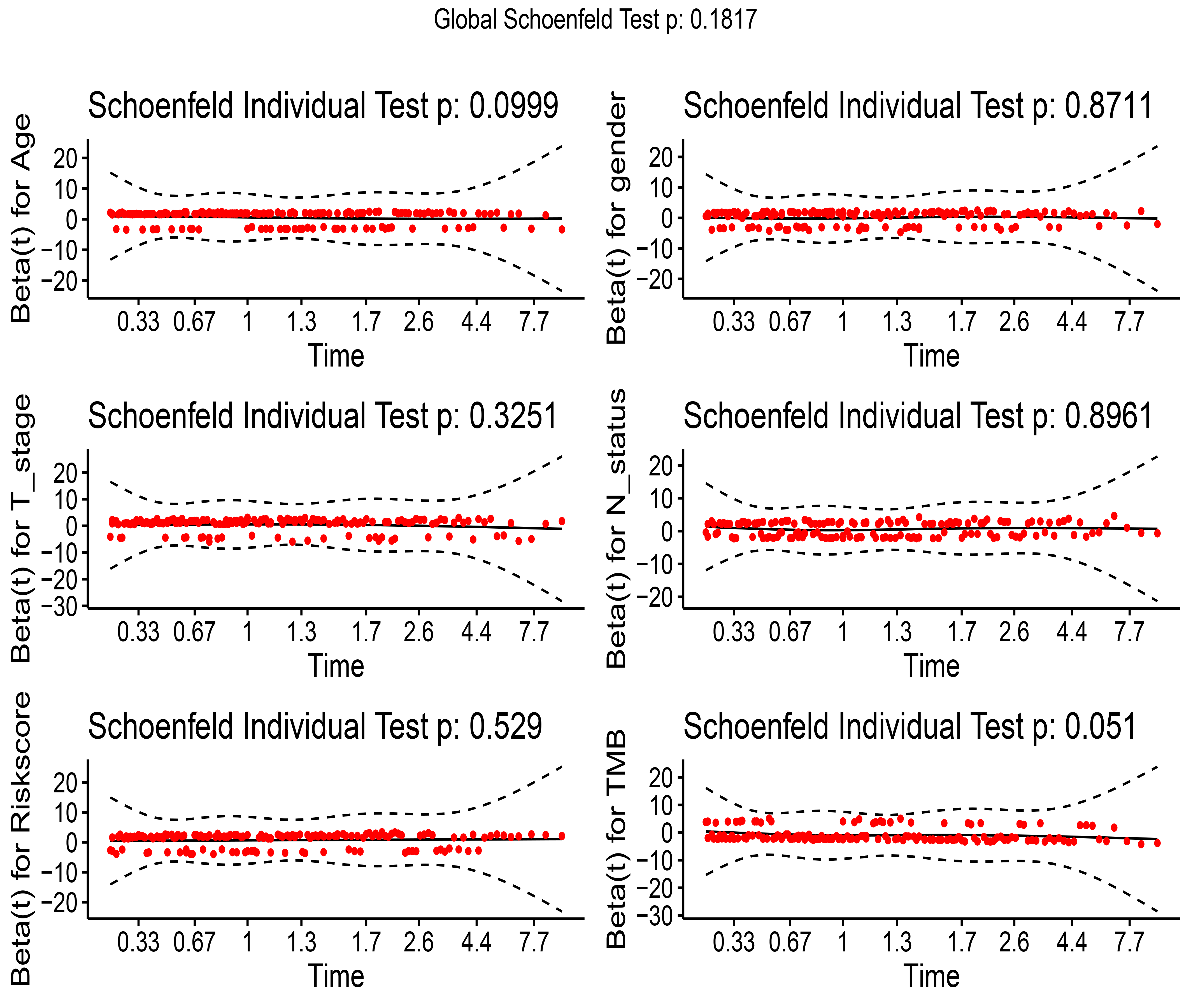

Supplement: Supplementary Figure 8 — The Schoenfeld Individual Test of six clinical characters, including age (A), gender (B), T, stage (C), N status (D), TIPS (E) and TMB (F). (TIPS, TP53-associated immune prognostic signature; TMB: tumor mutation burden). [file Image_8.tif]

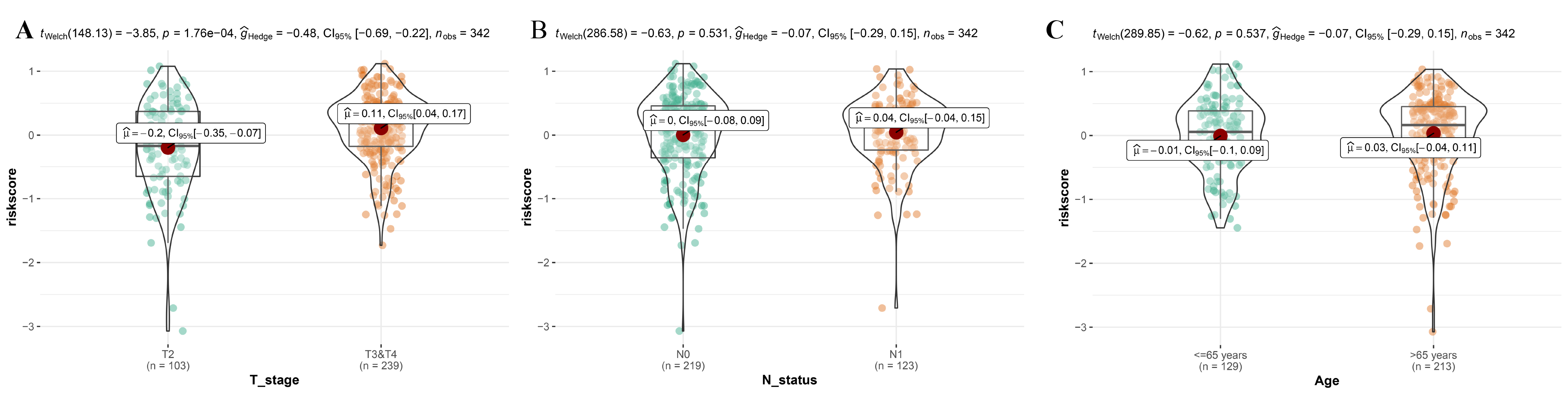

Supplement: Supplementary Figure 9 — Show the distribution of the risk score in different T stage (A), N status (B) and age (C) in TCGA cohort. (TCGA, The Cancer Genome Atlas). [file Image_9.tif]
